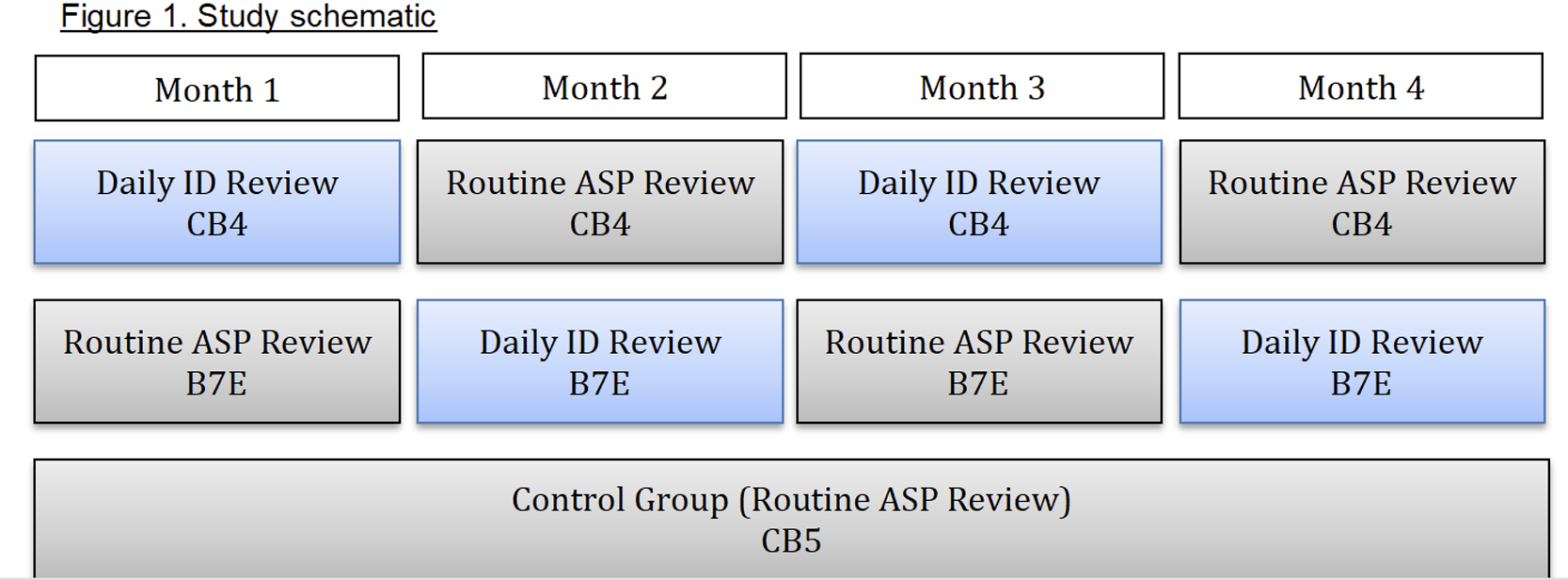# 120 Assessing Clostridioides difficile Survival and Disinfectant Performance on Mattress Cover Materials in Healthcare Settings

**DOI:** 10.1017/ash.2026.10534

**Published:** 2026-06-23

**Authors:** Ann Palmer, Tyler Ackley, Maria Akiki

**Affiliations:** 1 Hartford Hospital/University of Connecticut School of Medicine; 2 Hartford Hospital; 3 University of Connecticut

## Abstract

**Background:** In response to mounting antibiotic resistance and overuse, antimicrobial stewardship continues to serve as a major evolving initiative within healthcare institutions. Handshake stewardship utilizes in-person feedback to help facilitate intervention communication, in hopes of fostering clinician-ASP relationships and providing real-time education. This “face-to-face” approach has been demonstrated to improve antibiotic days of therapy (DOT) while also being perceived by treating teams as more favorable when compared to written feedback. **Methods:** Integrated ID/Pharmacy rounds occurred daily Monday – Friday, excluding holidays during the months of December 2024 to March 2025. The current hospital admitting structure separates individual hospitalist teams and internal medicine teaching resident teams. Teaching and hospitalist teams each have their own designated floors. To assess the long-lasting impact of ongoing antimicrobial stewardship education and intervention, an alternating checker box design was utilized, where ID/pharmacy rounds alternated between both designated teaching floors and used a hospitalist floor as control, where routine ASP review occured daily. Primary endpoints were antibiotic discontinuation, antibiotic deescalation and intravenous to oral conversion. Patients with an Infectious Diseases consultation were excluded. **Results:** Primary endpoints were antibiotic discontinuation, antibiotic deescalation and intravenous to oral conversion. Secondary endpoints included impact on length of stay and associated hospital acquired infections (C difficile). Overall: 196/388 patients (50.5%). Hospitalist control floor: 69/190 (36.3%). Teaching: 127/198 (64.6%); for the active teaching rounding group (75/106 (70.8%)) vs ASP education only (52/92 (57.6%)). For Hospitalist control floor vs teaching floor: p < 0.00001. Active teaching rounds vs ASP education: p = 0.037 **Conclusion:** Active participation and strong presence of ID pharmacy and ID physicians in daily medicine rounds, posiively impacts antibiotic utilization. It secures a direct line of communication and fosters strong relationships between departments, that ultimately benefits patient care, reduces lenght of stay and optimizes antibiotic utilization in terms of deescalation, duration of treatment and targeted antibiotic selection. Limitations include low availability of ID physicians for daily rounds, limited number of ID pharmacists for dailys ASP review and occasional disregard of recommendations by medicine teams.

**Figure:**